# Analysis of common respiratory pathogens and epidemiological trends during peak influenza seasons in Tengzhou

**DOI:** 10.1186/s12879-025-11146-4

**Published:** 2025-07-01

**Authors:** Huiping Ma, Jinghai Hou, Jing Wang, Zhaolin Yuan, Sijin Man

**Affiliations:** 1 Department of Laboratory Medicine, Tengzhou Central People’s Hospital, Tengzhou, Shandong 277599 P. R. China; 2 Department of Laboratory Medicine, Taierzhuang District People’s Hospital, Taierzhuang, Zaozhuang, Shandong 277400 P. R. China

**Keywords:** Respiratory pathogens, Respiratory infections, Age, Seasonal influenza, Epidemiology, Public Health

## Abstract

**Objective:**

To analyze the etiology and epidemiological characteristics of respiratory infections during the winter season in the Tengzhou region and provide localized data to support public health measures.

**Methods:**

A retrospective study was conducted on patients diagnosed with acute upper respiratory tract infections (ARTIs) between November 2023 and January 2024 at Tengzhou Central People's Hospital and Tengzhou Maternal and Child Health Hospital. Pathogens analyzed included influenza A (Flu A), influenza B (Flu B), respiratory syncytial virus (RSV), adenovirus (ADV), *Mycoplasma pneumoniae* (MP), and human rhinovirus (HRV). Statistical analyses were performed to evaluate infection patterns and their associations with age and other variables.

**Results:**

A total of 4,869 specimens were collected, with 2,307 cases (47.4%) identified as single- pathogen infections, 272 cases (10.9%) as dual-pathogen infections, and 37 cases (1.5%) as infections with three or more pathogens. The detection rates differed significantly between the < 14-year-old and ≥ 14-year-old groups (χ^2^ = 13.87, *P* < 0.05). Flu A was the most frequently detected pathogen (1,340 cases, 27.5%), followed by RSV (251 cases, 10.1%), ADV (241 cases, 9.7%), MP (223 cases, 4.6%), HRV (113 cases, 4.5%), and Flu B (139 cases, 2.9%). Detection rates for these six pathogens varied significantly between the < 14-year-old and ≥ 14-year-old groups (*P* < 0.05). Among the 1,340 Flu A-positive cases, 543 (40.5%) were H3N2-positive, accounting for 11.2% of all specimens. One H1N1 case was detected, co-infected with H3N2, and no H7N9 cases were found.

**Conclusions:**

This regional surveillance study highlights age-related differences in respiratory pathogen circulation in Tengzhou during the winter season. The findings support the need for continued seasonal monitoring and targeted prevention efforts.

## Introduction

Acute respiratory tract infections (ARTIs) remain a global public health concern, placing considerable strain on healthcare systems in both developed and developing countries [1, 2]. Viral agents such as influenza viruses, respiratory syncytial virus (RSV), adenovirus (ADV), and human rhinovirus (HRV) are the most common pathogens associated with ARTIs [3, 4]. Their genetic variability, transmissibility, and age-specific impacts make them a continuous challenge to surveillance and clinical management [5–7].

Regional and seasonal differences in virus circulation necessitate ongoing surveillance to guide prevention and control strategies [8]. Influenza, in particular, remains a research focus due to its antigenic drift and shift, which complicate outbreak prediction and vaccine formulation [9, 10]. Additionally, vulnerable populations such as children, the elderly, and immunocompromised individuals are disproportionately affected by RSV, ADV, and other viruses [11–13].

However, limited data are available on respiratory pathogen trends in many medium-sized cities in China, including Tengzhou [14]. Local surveillance studies are critical to informing timely public health responses, especially during peak winter seasons when healthcare systems are under strain [15, 16]. This study aimed to analyze the seasonal detection rates and age-group distributions of major respiratory viruses during the 2023–2024 winter season in Tengzhou, based on data collected from two major hospitals in the region.

## Materials and methods

### Sample sources

Specimens were collected from outpatient and inpatient departments of Tengzhou Central People's Hospital and Tengzhou Maternal and Child Health Hospital. The outpatient population primarily included individuals presenting with acute respiratory symptoms such as fever, sore throat, and cough, similar to patients seen in general practitioner settings. The study included throat swabs from patients diagnosed with acute upper respiratory tract infections over a three-month period, with a total of 4869 samples collected. Of these, 2490 specimens underwent combined detection for six respiratory pathogens, including influenza viruses (Flu A and Flu B), ADV, RSV, HRV, and MP. Additionally, 2379 samples were subjected to nucleic acid testing for Flu A, Flu B, and MP. Participants were divided into two age groups: children under 14 years and individuals aged 14 years and older, with ages ranging from 1 day to 104 years. Ethical approval for the study was obtained from the Ethics Committee of Tengzhou Central People's Hospital.

### Instruments and reagents

The following reagents and instruments were used for nucleic acid detection:

#### Reagents

Six Respiratory Pathogens Nucleic Acid Detection Kit (PCR-Fluorescent Probe Method, Sansure Biotech, Batch No. 20213400256); Nucleic Acid Extraction or Purification Reagent (Magnetic Bead Method, Daan Gene, Batch No. 202312009); Influenza A Virus Nucleic Acid Detection Kit (PCR-Fluorescent Probe Method, Batch No. 202401001); Influenza B Virus Nucleic Acid Detection Kit (PCR-Fluorescent Probe Method, Batch No. 202401001); *Mycoplasma pneumoniae* Nucleic Acid Detection Kit (PCR-Fluorescent Probe Method, Batch No. 202310007); Human H7N9 Avian Influenza Virus RNA Detection Kit (Fluorescent PCR Probe Method, Batch No. 202309001); Seasonal Influenza H3N2 Subtype Nucleic Acid Detection Kit (PCR-Fluorescent Probe Method, Batch No. 20231226); Influenza A H1N1 (2009) RNA Detection Kit (PCR-Fluorescent Probe Method, Batch No. 202310001).

#### Instruments

Fully Automated Nucleic Acid Extraction and Fluorescence PCR Analysis System (Anadas9850).

### Specimen collection

Nasopharyngeal swab samples were collected and stored in 6 mL viral transport medium. Samples were temporarily stored at 4 °C and transported to the molecular biology laboratory for analysis within 24 h. Specimens that could not be immediately tested or required further subtyping were stored at -70 °C or below.

### Detection methods

Nucleic acid detection for respiratory pathogens, including Flu A, Flu B, MP, ADV, RSV, HRV, and H7N9, was conducted using PCR fluorescence probe-based kits provided by Sansure Biotech and Daan Gene. All procedures were performed in strict accordance with the manufacturers’ protocols.

### Statistical analysis

Statistical analysis was performed using SPSS software version 19.0. Descriptive statistics were expressed as percentages, and chi-square tests (χ^2^) were applied for comparison. A *P*-value of < 0.05 was considered statistically significant.

## Results

### Detection of six respiratory pathogens

Among 4869 specimens, 47.4% showed single-pathogen infections, 10.9% dual infections, and 1.5% triple or more pathogens. Detection was significantly higher in the < 14-year-old group (χ^2^ = 42.2, *P* < 0.05). See Table 1 for detailed breakdown.

**Table 1:** Positive Rate of Viral Infections from November 2023 to January 2024

Date Infection Type	November 2023		December 2023		January 2024		Total
	< 14 years	≧14 years	< 14 years	≧14 years	< 14 years	≧14 years	
	Positive Rate (%)	Positive Rate (%)	Positive Rate (%)	Positive Rate (%)	Positive Rate (%)	Positive Rate (%)	Positive Rate (%)
Single pathogen	65.2 (234/359)	24.0 (66/275)	71.1 (923/1297)	30.2(255/846)	41.0 (567/1383)	37.0 (262/709)	47.4 (2307/4869)
Dual pathogen	7.7 (26/338)	4.3 (4/93)	11.7 (72/616)	2.8 (9/325)	17.2 (127/737)	8.9 (34/381)	10.9 (272/2490)
Three or more pathogens	0.0 (0/338)	0.0 (0/93)	1.1 (7/616)	0.0 (0/325)	3.3 (24/737)	1.6 (6/381)	1.5 (37/2490)

## Detection rates of single respiratory pathogen infections

Flu A was the most common pathogen (27.5%), followed by RSV (10.1%), ADV (9.7%), *Mycoplasma pneumoniae* (4.6%), HRV (4.5%), and Flu B (2.9%). All showed significant differences between age groups (*P* < 0.05), with children < 14 years consistently showing higher detection rates. See Table 2.

**Table 2:** Positive Rates of Various pathogens from November 2023 to January 2024 [%(Cases/Total)]

Date Pathogen	November 2023		December 2023		January 2024		Total
	< 14 years	≧14 years	< 14 years	≧14 years	< 14 years	≧14 years	
	Positive Rate (%)	Positive Rate (%)	Positive Rate (%)	Positive Rate (%)	Positive Rate (%)	Positive Rate (%)	Positive Rate (%)
FluA	30.4 (109/359)	19.6 (54/275)	47.1 (611/1297)	26.8 (227/846)	7.8 (149/1383)	26.8 (190/709)	27.5 (1340/4869)
FluB	0.0 (0/359)	1.5 (4/275)	0.4 (6/1297)	0.7 (6/846)	4.9 (95/1383)	3.9 (28/709)	2.9 (139/4869)
MP	12.5 (45/359)	1.1 (3/275)	6.3 (82/1297)	0.6 (5/846)	6.1 (77/1383)	1.6 (11/709)	4.6 (223/4869)
RSV	5.9 (20/338)	2.2 (2/93)	12.3 (76/616)	1.8 (6/325)	12.4 (138/737)	2.4 (9/381)	10.1 (251/2490)
ADV	8.9 (30/338)	3.2 (3/93)	18.7 (115/616)	0.0 (0/325)	4.3 (84/737)	2.4 (9/381)	9.7 (241/2490)
HRV	8.9 (30/338)	0.0 (0/93)	5.4 (33/616)	3.4 (11/325)	2.2 (24/737)	3.9 (15/381)	4.5 (113/2490)
Total	65.2 (234/359)	24.0 (66/275)	71.2 (923/1297)	30.1 (255/846)	41.0 (567/1383)	36.9 (262/709)	47.4 (2307/4869)

### Detection rates of co-infections with two respiratory pathogens

Among 2,490 samples tested, 272 dual-pathogen co-infections were detected (10.9%). The detection rate was significantly higher in children under 14 years old (χ^2^ = 29.97, *P* < 0.05), though no statistically significant variation was observed across months (*P* > 0.05).

For detailed age- and time-specific patterns, refer to Table 3.

**Table 3:** Positive Rates of Dual Respiratory pathogen Infections from November 2023 to January 2024 [%(Cases/Total)]

Date Pathegens	November 2023		December 2023		January 2024	
	< 14 years	≥ 14 years	< 14 years	≥ 14 years	< 14 years	≥ 14 years
	Positive Rate (%)	Positive Rate (%)	Positive Rate (%)	Positive Rate (%)	Positive Rate (%)	Positive Rate (%)
MP + ADV	1.5 (5/338)	0.0 (0/93)	1.5 (9/616)	0.0 (0/325)	3.1 (23/737)	0.8 (3/381)
MP + HRV	0.6 (2/338)	0.0 (0/93)	0.9 (6/616)	0.0 (0/325)	1.2 (9/737)	0.3 (1/381)
RSV + FluA	0.9 (3/338)	0.0 (0/93)	0.6 (4/616)	0.0 (0/325)	0.7 (5/737)	1.0 (4/381)
RSA + HRV	0.3 (1/338)	0.0 (0/93)	0.5 (3/616)	0.0 (0/325)	1.5 (11/737)	0.8 (3/381)
RSV + ADV	0.0 (0/338)	0.0 (0/93)	1.5 (9/616)	0.9 (3/325)	2.0 (15/737)	0.0 (0/381)
ADV + FluA	1.8 (6/338)	1.0 (1/93)	1.6 (10/616)	0.0 (0/325)	0.3 (2/737)	0.3 (1/381)
ADV + HRV	0.6 (2/338)	0.0 (0/93)	1.3 (8/616)	0.3 (1/325)	2.2 (16/737)	0.3 (1/381)
HRV + FluA	0.3 (1/338)	0.0 (0/93)	0.6 (4/616)	1.2 (4/325)	0.0 (0/737)	1.3 (5/381)
MP + FluA	1.8 (6/338)	2.2 (2/93)	2.3 (14/616)	0.3 (1/325)	0.5 (4/737)	1.6 (6/381)
MP + FluB	0.0 (0/338)	0.0 (0/93)	0.3 (2/616)	0.0 (0/325)	0.9 (7/737)	0.5 (2/381)
MP + RSV	0.0 (0/338)	0.0 (0/93)	0.3 (2/616)	0.0 (0/325)	1.5 (11/737)	0.8 (3/381)
FluA + FluB	0.0 (0/338)	1.0 (1/93)	0.0 (0/616)	0.0 (0/325)	0.0 (0/737)	1.3 (5/381)
ADV + FluB	0.0 (0/338)	0.0 (0/93)	0.0 (0/616)	0.0 (0/325)	1.9 (14/737)	0.5 (2/381)
HRV + FluB	0.0 (0/338)	0.0 (0/93)	0.0 (0/616)	0.0 (0325)	0.3 (2/737)	0.0 (0/381)
RSV + FluB	0.0 (0/338)	0.0 (0/93)	0.0 (0/616)	0.0 (0/325)	0.8 (6/737)	0.5 (2/381)
Total	7.7 (26/338)	4.3 (4/93)	11.7 (72/616)	2.8 (9/325)	17.2 (127/737)	8.9 (34/381)

### Subtyping of Flu A positive patients

Among 1,340 Flu A-positive cases, 40.5% were H3N2. One H1N1 co-infection was identified; no H7N9 cases were detected. H3N2 was significantly more common in children (χ^2^ = 13.04, *P* < 0.05). See Table 4.

**Table 4:** Positive Rates of Flu A and Subtypes from November 2023 to January 2024 [%(Cases/Total)]

Date Virus	November 2023		December 2023		January 2024		Total
	< 14 years	≧14 years	< 14 years	≧14 years	< 14 years	≥ 14 years	
	Positive Rate (%)	Positive Rate (%)	Positive Rate (%)	Positive Rate (%)	Positive Rate (%)	Positive Rate (%)	Positive Rate (%)
Flu A	30.4 (109/359)	19.6 (54/275)	47.1 (611/1297)	26.8 (227/846)	7.8 (149/1383)	26.8 (190/709)	27.5 (1340/4869)
H1N1	0.0 (0/359)	0.0 (0/275)	0.0 (0/1297)	0.0 (0/846)	0.0 (0/1383)	0.1 (1/709)	0.02 (1/4869)
H3N2	12.0 (43/359)	7.3 (20/275)	14.2 (184/1297)	18.8 (159/846)	5.3 (73/1383)	9.0 (64/709)	11.2 (543/4869)
H7N9	0.0 (0/359)	0.0 (0/275)	0.0 (0/1297)	0.0 (0/846)	0.0 (0/1383)	0.0 (0/709)	0.0 (0/4869)

2.5 Distribution of H3N2 Influenza A Cases by Gender and AgeOf 543 H3N2 cases, 55.2% occurred in children and 31.3% in adults ≥ 60 years. No significant gender difference was observed (χ^2^ = 2.55, *P* = 0.11). See Table 5 for details.

**Table 5:** Gender and Age Distribution of H3N2 Influenza A Patients (Cases, %)

Age (years)	Cases (%)	Female	Male
< 14	300 (55.2)	130 (62.8)	170 (50.6)
14–59	73 (13.5)	32 (15.5)	41 (12.2)
≧60	170 (31.3)	45 (21.7)	125 (37.2)
Total	543 (100.0)	207 (38.1)	336 (61.9)

### Detection rate of H1N1

**Fig. 1:**
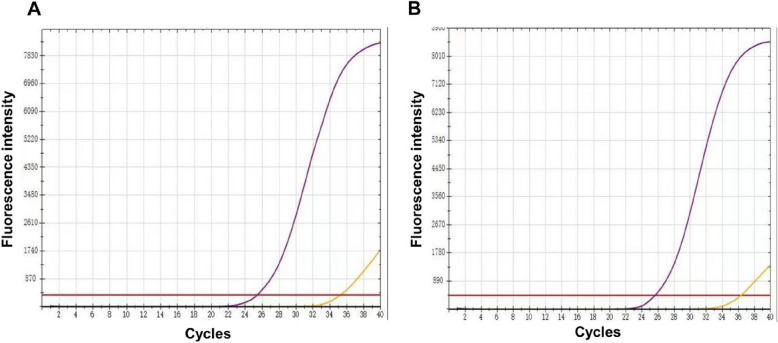
**A** The purple curve represents the N1( +) reaction curve, while the yellow curve represents the internal control curve. **B** The purple curve represents the H1( +) reaction curve, while the yellow curve represents the internal control curve

As shown in Table 4, one H1N1-positive case was identified in January 2024. This case was a co-infection with the H3N2 subtype. The fluorescence curves for this case are presented in Figs. 1

## Discussion

Respiratory infections in winter are predominantly caused by viruses, with pathogen detection rates influenced by age, season, and geographic factors [17]. In this study, 2307 single-pathogen infections were identified (47.4%), lower than the 64.74% reported by Li Xiaojun et al. [18], possibly due to regional and demographic differences. The top three detected pathogens were Flu A (27.5%), RSV (10.1%), and ADV (9.7%), whereas the 2021 Chinese CDC report identified HRV (16.7%) as more prevalent than RSV [19], indicating lower HRV activity in our region. For adults (≥ 14 years), HRV ranked higher than RSV, differing from findings by Wan Yiqiu et al. [21]. Additionally, RSV and HRV detection rates in our study were below national averages for the same winter period [19], likely reflecting regional variations in virus circulation and diagnostic strategies.Children under 14 years showed higher detection rates, which may be attributed to immature immune systems and behavioral factors such as poor hygiene and frequent close contact in school settings [19, 22].

Dual-pathogen co-infections were found in 10.9% of cases, more than Wu Weiwei’s report (7.4%) [20] but less than that of Zhu Lingping (29.8%) [23]. These occurred more frequently in children (13.3%) than in adults (5.9%), with RSV and ADV being the most commonly involved. Although triple-pathogen co-infections (1.5%) were observed, this likely underestimates the true burden due to the limited scope of tested pathogens. Viruses not included in the panel, such as seasonal coronaviruses and metapneumoviruses, may have contributed.

Among 1340 Flu A-positive cases, 543 (40.5%) were H3N2, peaking in December. No significant gender differences were found, but individuals aged ≥ 60 years had higher H3N2 positivity. These findings underscore the need for targeted protection of older adults through vaccination, hand hygiene, and avoidance of high-risk environments [24, 25].

A rare co-infection with H3N2 and H1N1 was identified in one patient, consistent with the local dominance of H3N2. The near-absence of H1N1 contrasts with surveillance data from Beijing [26] and from Europe and the United States, where co-circulation of H1N1pdm09 and H3N2 was more common during the same season [27, 28]. This may reflect differences in regional viral dynamics and immunity profiles.

Mycoplasma pneumoniae (MP) accounted for 4.6% of positive cases, mostly in children. Although often mild, MP can prolong illness and co-infect with viruses, complicating clinical management [10, 18].

This study represents a descriptive, cross-sectional analysis of respiratory pathogens during the 2023–2024 winter in Tengzhou. Due to the lack of patient-level metadata such as vaccination status or comorbidities, we were unable to assess individual risk factors for influenza or other infections. Findings should be interpreted in the context of the study’s limited temporal and geographic scope, which may not reflect broader national trends. Limitations include: (1) data from only two hospitals over a single winter season, (2) a restricted testing panel excluding SARS-CoV-2 and other pathogens, (3) lack of individual clinical or vaccination information, and (4) absence of outcome data. Future research should incorporate a broader pathogen panel, include individual-level risk variables, and explore clinical outcomes to better inform public health strategies.

## Data Availability

Data is provided within the manuscript or supplementary information files. Data are available from the authors upon reasonable request.
